# Divergent Inhibitor Susceptibility among Airway Lumen-Accessible Tryptic Proteases

**DOI:** 10.1371/journal.pone.0141169

**Published:** 2015-10-20

**Authors:** Shilpa Nimishakavi, Wilfred W. Raymond, Dieter C. Gruenert, George H. Caughey

**Affiliations:** 1 Cardiovascular Research Institute, University of California San Francisco, San Francisco, California, United States of America; 2 Veterans Affairs Medical Center, San Francisco, California, United States of America; 3 Department of Otolaryngology-Head and Neck Surgery, University of California San Francisco, San Francisco, California, United States of America; 4 Eli and Edythe Broad Center for Regenerative Medicine and Stem Cell Research, University of California San Francisco, San Francisco, California, United States of America; 5 Helen Diller Family Comprehensive Cancer Center, University of California San Francisco, San Francisco, California, United States of America; 6 Institute for Human Genetics, University of California San Francisco, San Francisco, California, United States of America; 7 Department of Pediatrics, University of Vermont College of Medicine, Burlington, Vermont, United States of America; 8 Department of Medicine, University of California San Francisco, San Francisco, California, United States of America; University of Pittsburgh, School of Medicine, UNITED STATES

## Abstract

Tryptic serine proteases of bronchial epithelium regulate ion flux, barrier integrity, and allergic inflammation. Inhibition of some of these proteases is a strategy to improve mucociliary function in cystic fibrosis and asthmatic inflammation. Several inhibitors have been tested in pre-clinical animal models and humans. We hypothesized that these inhibitors inactivate a variety of airway protease targets, potentially with bystander effects. To establish relative potencies and modes of action, we compared inactivation of human prostasin, matriptase, airway trypsin-like protease (HAT), and β-tryptase by nafamostat, camostat, bis(5-amidino-2-benzimidazolyl)methane (BABIM), aprotinin, and benzamidine. Nafamostat achieved complete, nearly stoichiometric and very slowly reversible inhibition of matriptase and tryptase, but inhibited prostasin less potently and was weakest versus HAT. The IC_50_ of nafamostat’s leaving group, 6-amidino-2-naphthol, was >10^4^-fold higher than that of nafamostat itself, consistent with suicide rather than product inhibition as mechanisms of prolonged inactivation. Stoichiometric release of 6-amidino-2-naphthol allowed highly sensitive fluorometric estimation of active-site concentration in preparations of matriptase and tryptase. Camostat inactivated all enzymes but was less potent overall and weakest towards matriptase, which, however was strongly inhibited by BABIM. Aprotinin exhibited nearly stoichiometric inhibition of prostasin and matriptase, but was much weaker towards HAT and was completely ineffective versus tryptase. Benzamidine was universally weak. Thus, each inhibitor profile was distinct. Nafamostat, camostat and aprotinin markedly reduced tryptic activity on the apical surface of cystic fibrosis airway epithelial monolayers, suggesting prostasin as the major source of such activity and supporting strategies targeting prostasin for inactivation.

## Introduction

Prostasin, matriptase, airway trypsin-like protease, and mast cell β-tryptase are trypsin-like proteases associated with airway mucosa. The present study profiles inhibitor susceptibility and mechanisms of inactivation of purified forms of these proteases. Prostasin (product of *PRSS8*), also called channel-activating protease, is a type I transmembrane protease that is anchored to airway cell surfaces via glycosylphosphatidyl inositol [1,2]. By activating the epithelial sodium channel (ENaC), prostasin increases Na^+^ reabsorption by luminal epithelium, thereby regulating hydration. Cell surface-prostasin resists inactivation by endogenous anti-proteases [3]. Knockdown of expression [4] and pharmacological inhibition decrease ENaC-mediated Na^+^ flux and are therapeutic strategies in cystic fibrosis [5,6], which may be associated with overactive ENaC. However, prostasin expression by alveolar epithelium is required for normal fluid clearance [7]. For proposed functions like regulating tight junctions and preserving epithelial barrier integrity [8], prostasin may depend on matriptase (product of *ST14*) [9,10]. In contrast to prostasin, matriptase is a type II transmembrane protease with non-catalytic extracellular domains and is regulated by hepatocyte growth factor activator inhibitor-1 [11]. Matriptase and prostasin activation may be co-dependent [10,12]. Therapeutic inhibition of matriptase has been proposed in conditions, like malignancy, in which it is overexpressed and may promote tumor cell tissue invasion [13]. On the other hand, matriptase is critical for skin and gut barrier function and is protective in models of colitis [14].

Less is known about airway trypsin-like protease (HAT in humans, product of *TMPRSS11D*) [15–17], which is a type II transmembrane protease. HAT is shed as an active, soluble protease into airways, where it promotes mucus production and is a target for inhibition in bronchitis [18]. β-tryptases (products of *TPSAB1* and *TPSB2*) are produced by epithelium-infiltrating mast cells in asthma [19], in which tryptase inactivation is potentially therapeutic [20]. β-tryptase assembles into a toroidal tetramer, which affords protection from proteinaceous inactivators of trypsin-like proteases. It is stored in secretory granules and released from stimulated mast cells. Secreted β-tryptases can stimulate epithelial cell growth, cytokine production, and recruitment of inflammatory cells [21,22].

The inhibitors analyzed in this work were selected for therapeutic potential as topical inhibitors of tryptic proteases in the airway lumen. Most of these compounds have been tested in preclinical models of human disease or in humans, although neither the therapeutically relevant target nor the potential bystander targets of these inhibitors is known with certainty. Two of the inhibitors, nafamostat and camostat, are guanidinobenzoates that may be cleaved by proteases and can be suicide inhibitors [23–25]. Nafamostat has been used clinically as an anticoagulant in humans, in whom the targets are thought to be proteases of the clotting cascade. It is an inhibitory substrate for pancreatic trypsin, with which it forms a stable acyl-enzyme intermediate [23], and it is a particularly potent inhibitor of mast cell tryptases [26]. Nafamostat reduces inflammation in rats with colitis and mice with experimental asthma, possibly by inactivating tryptases [27,28], and reduces tryptase-induced itching in mice [29]. Camostat, on the other hand, attenuates kidney fibrosis in a rat model of chronic renal failure [30]. Given to hypertensive rats, camostat reduces blood pressure and improves kidney function [31], possibly by targeting renal prostasin. When applied to airway mucosa, camostat durably inhibits ENaC in guinea pig trachea, enhances mucociliary clearance in sheep bronchi [6], and increases transepithelial nasal potential difference in humans with cystic fibrosis, by mechanisms speculated to involve inhibition of prostasin [32].

Benzamidine and BABIM, by contrast, are competitive, reversible inhibitors. Benzamidine is a general tryptic protease inhibitor that reduces tryptase-induced enhancement of muscle contraction in isolated bronchi [33]. BABIM is a bifunctional aromatic amidine that inhibits tryptase and trypsin [34,35]. In sheep, topical BABIM blocks asthma-like airway responses, including increased resistance in allergen-challenged airway, potentially by inhibiting tryptase [20]. Aprotinin is a proteinaceous general inhibitor of tryptic serine proteases that blocks the substrate-binding site. Because of its size, it is ineffective versus proteases with restricted active sites [36]. It has been used as a drug in humans to treat pancreatitis and to limit blood loss in surgery [37]. Clinical targets may include kallikreins and activated proteases associated with complement activation and hemostasis. Aprotinin and related inhibitors are proposed as inhaled therapeutic inhibitors in cystic fibrosis [5]. When applied intratracheally, aprotinin attenuates epithelial ion transport in guinea pigs [38], and when applied to human airway epithelial monolayers, aprotinin reduces ENaC function [6] and surface tryptic activity [3].

## Materials and Methods

### Sources of key reagents

**Fig 1:**
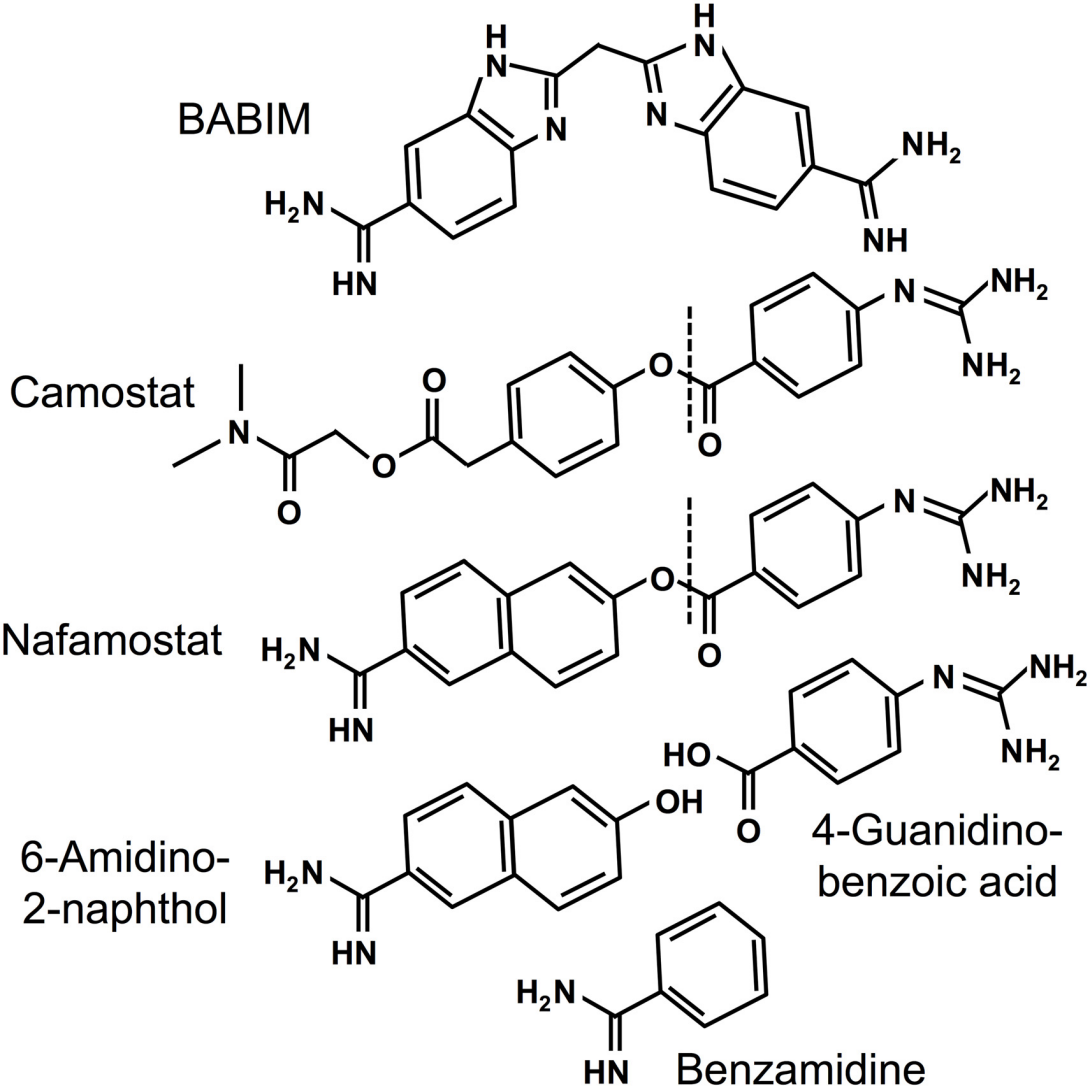
Structures of aromatic amidines and related compounds. All compounds have amidino and/or guanidino functional groups. BABIM is bifunctional but unhydrolyzable. Camostat and nafamostat share a cleavable guanidinobenzoyl moiety with potential to form an acyl intermediate involving the active site serine hydroxyl of serine proteases. The dotted lines indicate the site of cleavage. 6-Amidino-2-naphthol is nafamostat’s fluorescent leaving group generated by formation of the acyl intermediate, and itself inhibits tryptic serine proteases.

Recombinant human prostasin, matriptase and HAT, expressed as soluble catalytic domains, were obtained from R&D Systems (Minneapolis, MN). Human lung (β) tryptase was from EMD-Millipore (Billerica, MA). The tryptic peptidase substrates *t*-butyloxycarbonyl-L-Gln-L-Ala-L-Arg-4-nitroanilide (QAR) and N-(p-tosyl)-Gly-L-Pro-L-Lys-4-nitroanilide (GPK) were from Bachem Americas (Torrance, CA) and Sigma-Aldrich (St. Louis, MO), respectively. Protease inhibitors camostat mesylate and nafamostat mesylate were from Santa Cruz Biotechnology (Dallas, TX), as was nafamostat’s cleavage product 6-amidino-2-naphthol. Benzamidine, aprotinin, p-nitrophenyl-p’-guanidinobenzoate and bovine trypsin were from Sigma-Aldrich. BABIM was provided by Dr. Richard Tidwell as described previously [34]. Structures of chemical inhibitors used in this study are given in Fig 1.

### Determination of IC_50_

To compare potency of irreversible (or slowly reversible) inhibitors (for which *K*_i_ values may be inapplicable) with potency of competitive, reversible inhibitors, we determined the concentration of inhibitor that achieves 50% inactivation (IC_50_) of soluble forms of the following human airway tryptic proteases: prostasin, matriptase, HAT, and β-tryptase. IC_50_ was determined by assaying initial rates of hydrolysis of 1-mM QAR in 50-mM Tris-HCl (pH 7.6) containing 120-mM NaCl 0.05% Tween 20, and 100 μg/ml bovine lung heparin (Sigma-Aldrich) at 37°C over a range of inhibitor concentrations (shown in Table 1). Serial absorbance measurements detecting products of substrate hydrolysis were performed at 410 nm in wells of 96-well, flat-bottom, polystyrene Costar plates (Corning, Tewksbury, MA) in a kinetic, temperature-controlled spectrophotometer (Synergy 2, BioTek Instruments, Winooski, VT). Microplates were covered with TempPlate Optical Film (USA Scientific, Ocala, FL) during spectrophotometric measurements to minimize evaporation.

**Table 1:** Protease and inhibitor concentrations.

	HAT (0.72 nM)^a^	Matriptase (2.4 nM)^a^	Prostasin (80 nM)^a^	Tryptase (0.70 nM)^a^
**Aprotinin**	0.70–7000 nM	1.7–96 nM	0.80–320 nM	7.0–28000 nM
**BABIM**	0.7–350 nM	0.38–96 nM	13–1600 nM	0.7–5600 nM
**Benzamidine**	1.6–20 μM	0.24–1200 μM	1.6–20 μM	0.007–175 μM
**Camostat**	0.7–350 nM	3.8–12000 nM	13–3200 nM	0.044–350 nM
**Nafamostat**	1.4–4400 nM	0.0038–9.6 nM	8.0–8000 nM	0.014–3.5 nM
**6-Amidino-2-naphthol**	0.036–910 μM	0.15–480 μM	ND^b^	0.044–180 μM

^a^ Parentheses contain estimated protease concentration

^b^ Not done, due to insufficient inhibition

### Measurement of duration of inhibition by nafamostat

The durability of nafamostat inhibition was tested for the two proteases (matriptase and β-tryptase) for which inhibition was nearly stoichiometric. To permit assessment of reversibility, the proteases and inhibitors were combined in equimolar concentrations in the presence of substrate QAR under conditions in which enzyme activity was nearly but not completely inhibited. Recovery of activity was monitored over 4–5 hours and compared with activity of uninhibited enzyme.

### Assessment of product inhibition

Catalytic hydrolysis of nafamostat yields free 6-amidino-2-naphthol, which is an aromatic amidine related to several of the compounds tested in this study (see Fig 1) with potential to inhibit tryptic serine proteases. Thus, it could cause “product inhibition” and potentially account for prolonged inhibition of protease activity by nafamostat. To test this possibility, we used substrate QAR to compare the IC_50_ of nafamostat and 6-amidino-2-naphthol versus matriptase and β-tryptase.

### Active site titration of protease active sites using nafamostat

As a calibration control, bovine trypsin was active site-titrated by an established colorimetric method as described [39]. Briefly, 1- to 3-microliter aliquots of trypsin (30 mg/ml) were added to 0.5 ml of 1-mM p-nitrophenyl-p’-guanidinobenzoate in 0.1 M Tris-HCl (pH 8.2) containing 0.02 M CaCl_2_ in a quartz cuvette at 37°C while monitoring the “burst” increase in Absorbance at 405 nm on a Genesys 10S UV-Vis spectrophotometer equipped with a Peltier cuvette jacket (Thermo Fisher Scientific, Waltham, MA). The concentration of trypsin active sites was deduced using a molar extinction coefficient for 4-nitrophenol of 1.8 x 10^4^ Absorbance Units M^-1^ cm^-1^. The same preparation of trypsin then was active site-titrated by the more sensitive fluorometric approach using nafamostat. To establish the relationship between fluorescence emission (in mV) and concentration for 6-amidino-2-naphthol, which is the fluorescent leaving group generated by formation of the acyl intermediate by trypsin’s attack on nafamostat [23], serial dilutions of 6-amidino-2-naphthol in PBS were prepared. Fluorescence was measured using excitation and emission wavelengths of 320 nm and 490 nm, respectively, by injecting 0.1-ml aliquots into the 16-microliter flow cell of a Jasco FP-2020 Plus Fluorescence Detector (gain = 1000, attenuation = 8; Jasco Incorporated, Easton MD) at 0.1 ml/min to minimize bleaching. Flow was controlled by an AKTA Purifier 10 chromatography system (GE Healthcare Biosciences, Pittsburgh, PA). Fluorescence readings were blanked by injecting PBS. Molarity of active sites in solutions of trypsin was estimated and compared with values obtained by the colorimetric p-nitrophenyl-p’-guanidinobenzoate approach by adding aliquots of enzyme stock solutions to nafamostat in PBS and measuring increase in fluorescence. Active site concentrations in preparations of matriptase and β-tryptase were determined fluorometrically in similar fashion.

### Determination of specific activities

Standard assay conditions for active site-titrated enzymes were 1-mM QAR in 50-mM Tris-HCl (pH 8.8) containing 50-mM NaCl and 0.01% Tween 20 at 37°C for matriptase, and 1-mM GPK in PBS with 100 μg/ml bovine lung heparin (Sigma-Aldrich) for β-tryptase.

### Culturing human airway cells

Human CFBE41o- bronchial epithelial cells [40,41] were grown to confluence and differentiated in air-liquid interface culture as previously described [3]. Briefly, cells were seeded to confluence at 3x10^5^ cells/well onto Costar Transwell membranes (0.33 cm^2^, 0.4-μm pores; Corning; Lowell, MA) coated with bovine serum albumin (1 mg/ml; Invitrogen, Carlsbad, CA), human fibronectin (30 μg/ml; BD Biosciences; San Jose, CA), and bovine collagen type II (10 μg/ml; BD Biosciences) in LHC basal medium (Invitrogen) and grown in minimal essential medium with Earle’s salt supplemented with 10% fetal bovine serum, 4 mM L-glutamine, 100 U/ml penicillin G, and 300 μg/ml hygromycin at 37°C in a 5% CO_2_ incubator. Apical medium was removed 48 h after seeding to initiate air-interface culture. Basolateral medium was changed every 48 h for 12 days. Except as specified, culture media and supplements were from Invitrogen.

### Assay of protease activity on the surface of airway epithelial cell monolayers

Tryptic protease activity on the apical surface of confluent CFBE41o- bronchial epithelial cell monolayers was measured by methods previously described [3]. We employed these cells because they form differentiated, electrically resistant monolayers that contain apical prostasin and matriptase and surface aprotinin-inhibitable tryptic activity [3]. Briefly, cells were washed in PBS after 12 days of air-interface culture on Transwell inserts. The apical side of the monolayer was immersed in 0.2 ml of PBS alone, PBS containing tryptic substrate QAR (1 mM) alone as control, or PBS containing QAR plus inhibitor (0.1 mM aprotinin, 0.01 mM nafamostat, or 0.01 mM camostat). The basolateral surface was bathed in PBS alone. Tryptic activity in aliquots of apical conditioned medium was monitored for 5 h at 37°C by spectrophotometry at 410 nm using a Synergy 2 SL Microplate Reader (BioTek Instruments).

### Statistical methods

IC_50_ values and associated confidence intervals were determined using the Hill slope curve-fitting utility implemented in Prism (GraphPad Software, La Jolla, CA). Differences in surface tryptic activity of cells incubated in the presence of substrate alone versus substrate plus inhibitor were compared using 1-tailed Student’s t-tests.

## Results

### Inhibitor susceptibility of tryptic airway proteases

**Fig 2:**
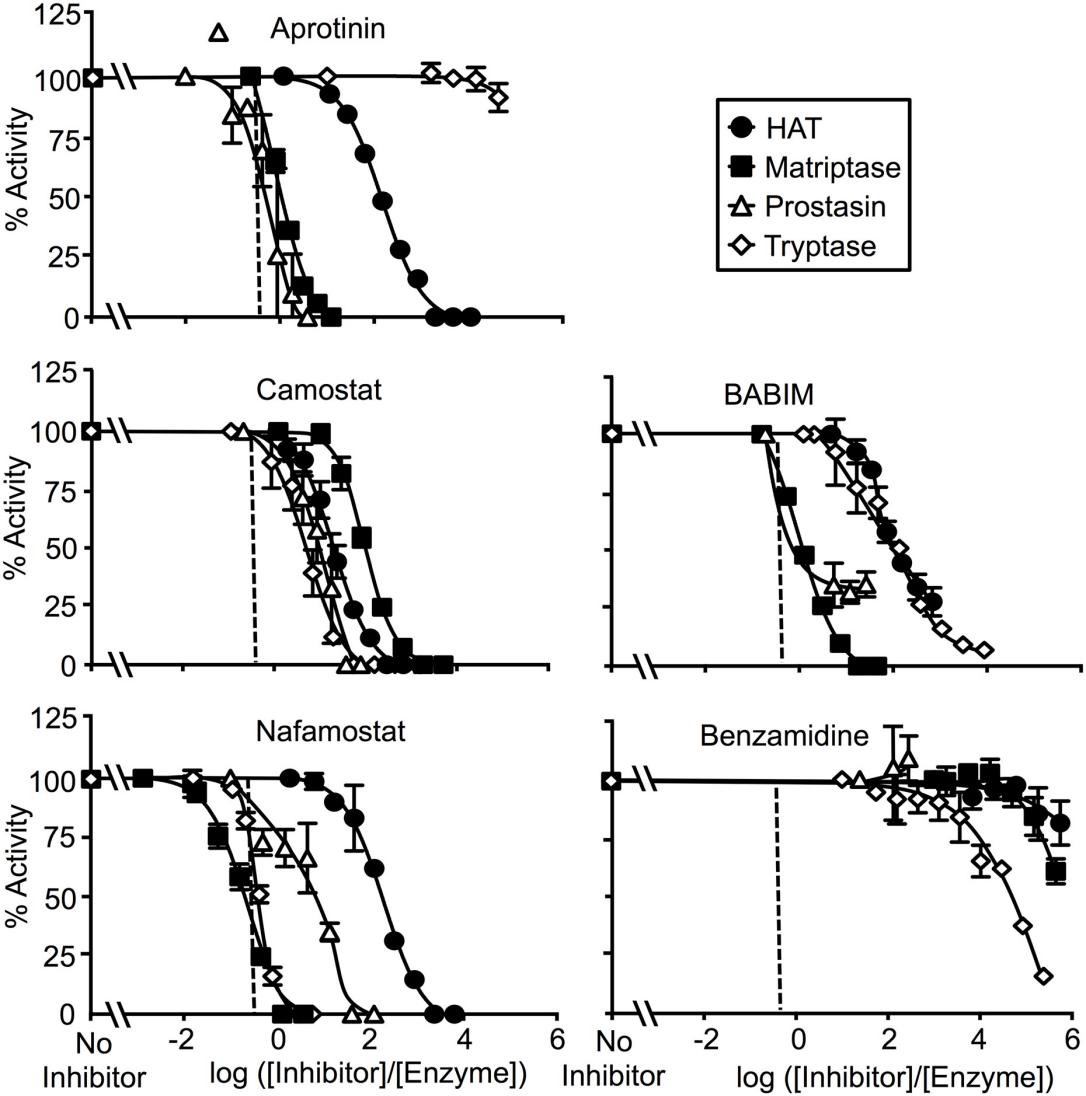
Comparison of inhibitor potencies. Data are expressed as percentage of activity relative to no-inhibitor control activity versus log_10_ ratios of inhibitor to protease concentration. The proteases tested are human airway trypsin-like protease (HAT), matriptase, prostasin and β-tryptase. The dashed vertical line marks the inhibitor/enzyme ratio at which 50% inhibition is predicted for inactivation with 1:1 stoichiometry. Aprotinin achieves near-stoichiometric inhibition of prostasin and matriptase, whereas nafamostat achieves near-stoichiometric inhibition of tryptase and matriptase. N = 3–4; error bars show ± S.D.

**Fig 3:**
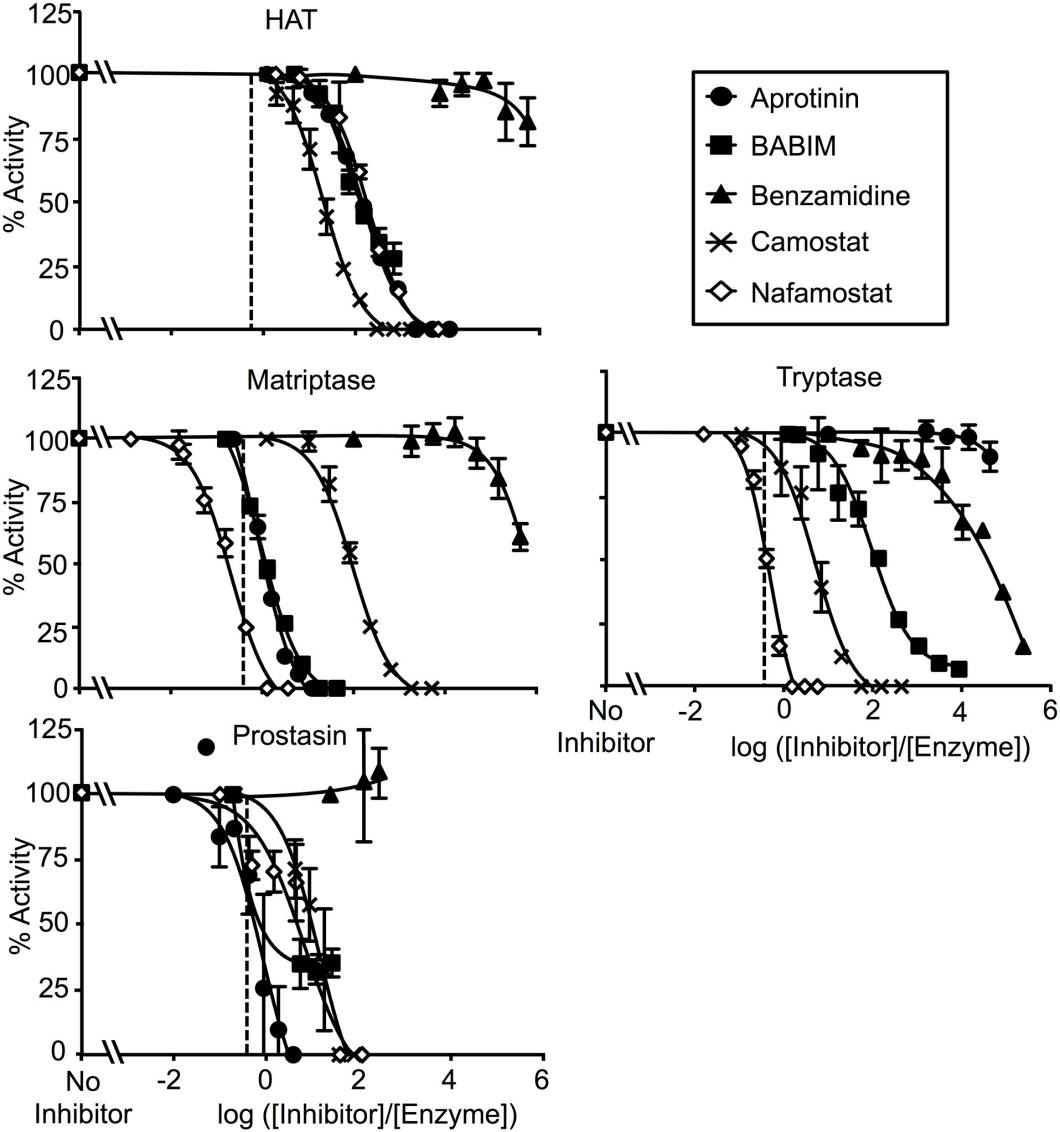
Comparison of enzyme susceptibility to inhibition. These panels display the data in Fig 2 to facilitate comparison of sensitivity of individual proteases to the inhibitors tested. N = 3–4; error bars show ± S.D.

The in vitro, steady state susceptibility of four human airway proteases (HAT, matriptase, prostasin and β-tryptase) to inactivation varied depending on the inhibitor studied. Fig 2 compares the potency of individual inhibitors versus the four proteases. Fig 3 compares the inhibitor susceptibility of individual proteases versus the five inhibitors. To facilitate comparison of potencies over a range of inhibitor and protease concentrations, the data are graphed as percentage of uninhibited enzyme activity versus log of the ratio of inhibitor concentration to starting concentration of active enzyme (log_10_ ([inhibitor]/[enzyme])). In such plots, activity is 0% when log_10_ ([inhibitor]/[enzyme]) = 0 for an inhibitor that inactivates a protease with full potency and 1:1 stoichiometry. The normalized IC_50_ for such an inhibitor would be log_10_ (0.5) = -0.3. The observed IC_50_ data are shown in Table 2.

**Table 2:** Normalized IC_50_ (ratio of inhibitor to protease concentration achieving 50% inhibition).

	HAT	Matriptase	Prostasin	Tryptase
**Aprotinin**	147 [134, 163]^a^	0.76 [0.59, 0.98]	0.86 [0.36, 2.02]	ND^b^
**BABIM**	71 [48, 105]	0.98 [0.87, 1.11]	ND	108 [78, 149]
**Benzamidine**	1.4e6 [2.0e5, 9.6e6]	1.1e6 [9.4e5, 1.2e6]	ND	4.7e4 [3.4e4, 6.5e4]
**Camostat**	20 [16, 24]	96 [80, 115]	14 [7.7, 25]	5.2 [3.7, 7.2]
**Nafamostat**	191 [154, 239]	0.21 [0.16, 0.26]	11 [5.6, 20]	0.44 [0.29, 0.67]
**6-Amidino-2-naphthol**	3.8e5 [1.6e5, 9.3e5]	6.0e4 [1.6e4, 2.2e5]	ND	1.2e4 [8.5e3, 1.7e4]

^a^Brackets contain lower and upper limits of 95% confidence intervals

^b^Not determined, due to insufficient inhibition

As revealed in Fig 2, nafamostat achieved complete and nearly 1:1 stoichiometric inactivation of matriptase and tryptase, but less potently inhibited prostasin and was weak versus HAT, for which ~10,000-fold excess of inhibitor over enzyme was required to achieve complete inhibition. For both matriptase and tryptase, nafamostat was the most potent of all anti-proteases studied. Camostat inactivated all enzymes but overall was substantially less potent than nafamostat, and exhibited the least ability of all of the inhibitors to discriminate between the tryptic airway proteases examined in this study. Although camostat, like nafamostat, is a guanidinobenzoate (see Fig 1), it was weakest towards matriptase whereas nafamostat was the strongest, suggesting that the leaving groups (prior to release) for these cleavable inhibitors are major determinants of specificity and potency. BABIM, which is an aromatic amidine but is not hydrolysable like nafamostat and camostat, was a complete and potent inhibitor of matriptase, but was less so for the other enzymes. Aprotinin exhibited nearly 1:1 stoichiometric inhibition of prostasin and matriptase, but was much weaker towards HAT and completely ineffective versus tryptase. Benzamidine was universally weak and did not fully inactivate any of the proteases at stoichiometries as high as 1,000,000:1.

### Inhibition by 6-amidino-2-naphthol does not explain high potency of nafamostat versus matriptase and β-tryptase

**Fig 4:**
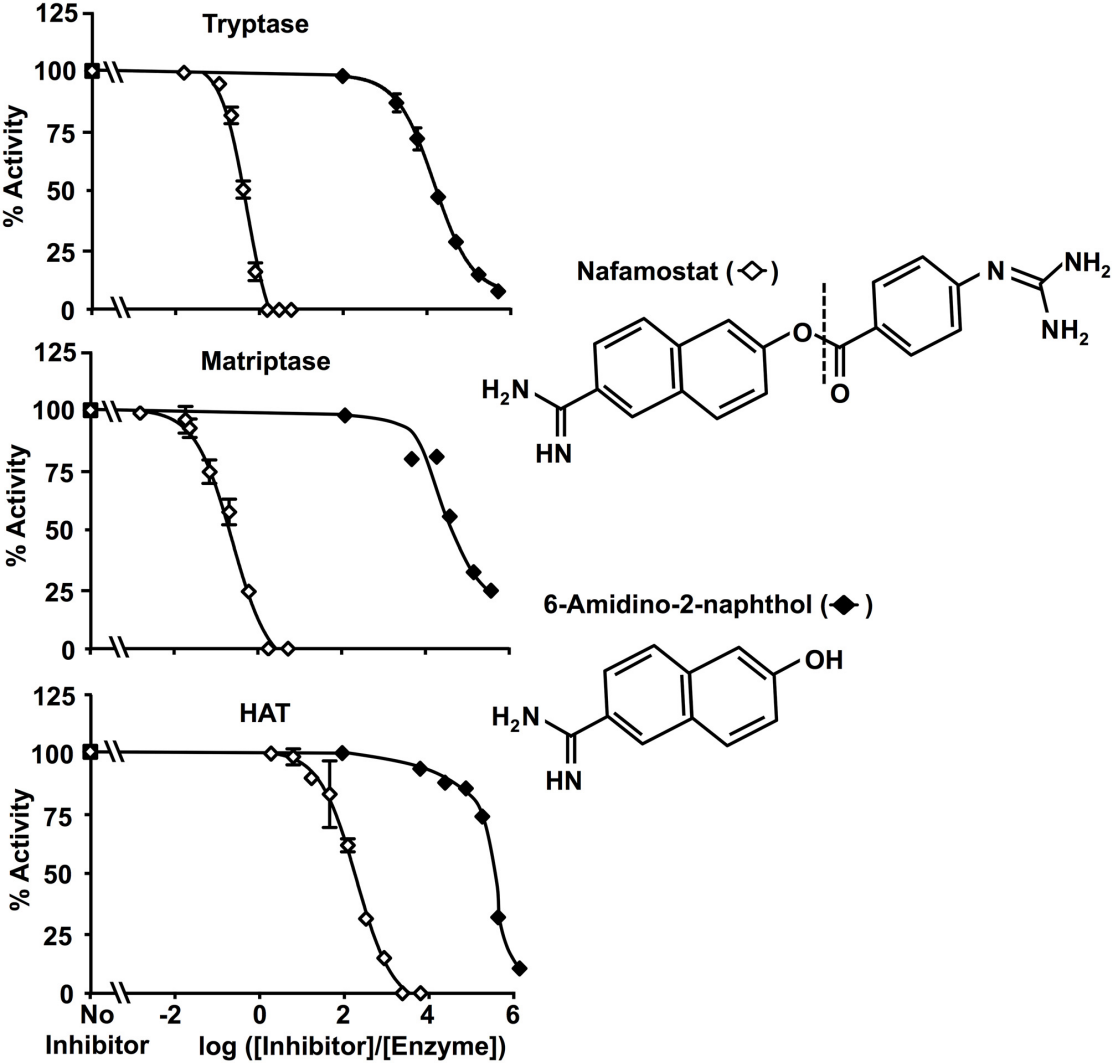
Product inhibition by 6-amidino-2-naphthol. These graphs compare inhibitory potency of nafamostat with that of its liberated cleavage product, 6-amidino-2-naphthol, versus matriptase and β-tryptase.

As shown in Fig 4, the IC_50_ of nafamostat’s leaving group, 6-amidino-2-naphthol, was >10^4^-fold higher for matriptase and β-tryptase than the IC_50_ of nafamostat itself. Although one potential mechanism of high potency of a cleavable inhibitor is generation of a leaving group that is a more potent competitive inhibitor than is the uncleaved parent compound, the very high IC_50_ of 6-amidino-2-naphthol relative to that of nafamostat itself suggests that this is not the case. Therefore, inhibition by the non-acyl component of hydrolyzed nafamostat does not explain nafamostat’s high potency upon cleavage by these enzymes and, given the very low rates of nafamostat turnover, 6-amidino-2-naphthol would be unlikely to achieve the concentrations needed to reduce matriptase and β-tryptase activity.

### Inhibition of matriptase and β-tryptase by nafamostat spontaneously reverses

**Fig 5:**
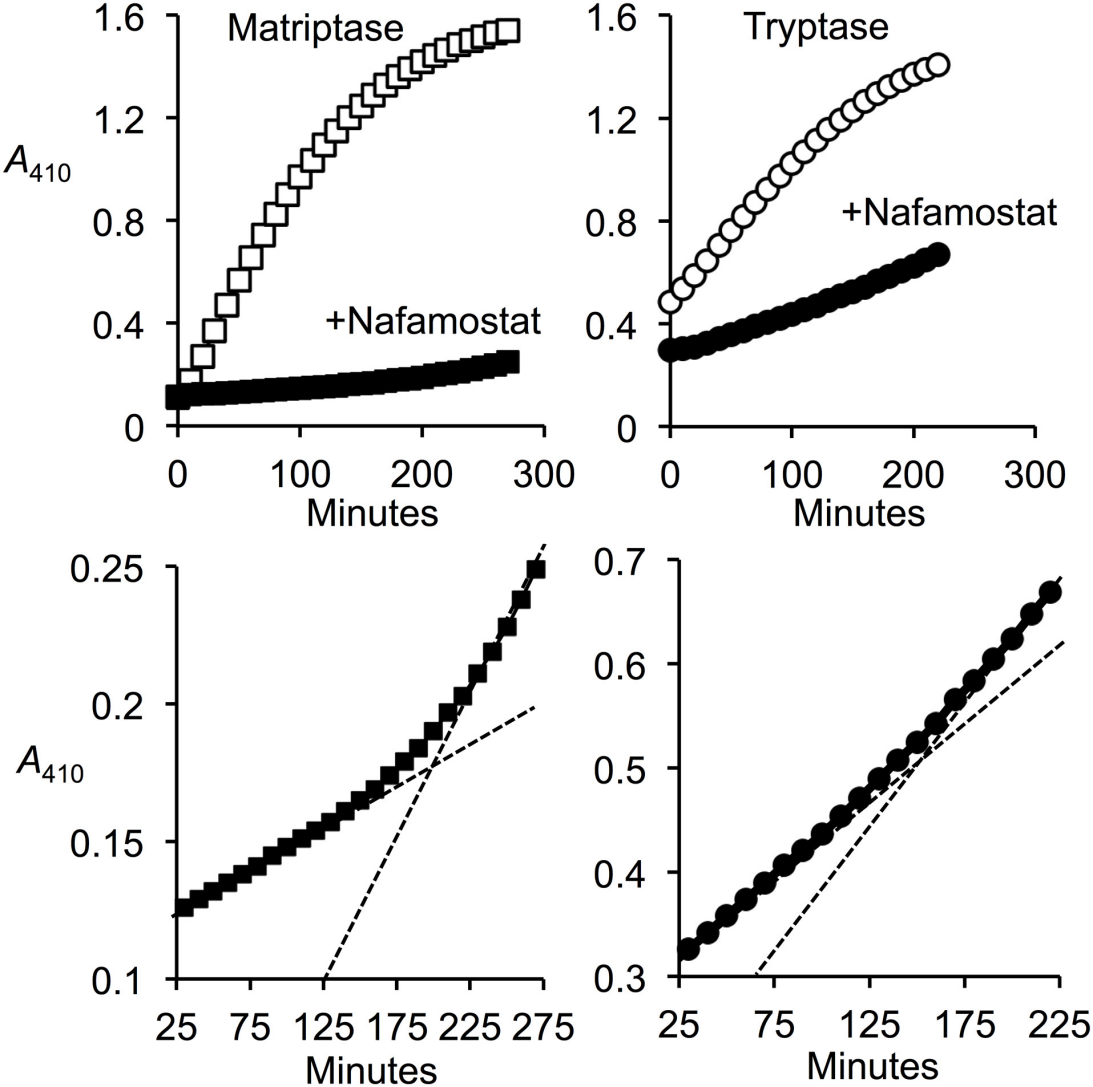
Recovery of matriptase and tryptase from inhibition by nafamostat. The upper panels compare cumulative hydrolysis of substrate QAR by matriptase and β-tryptase with and without addition of near-equimolar nafamostat. The bottom panels contain a subset of the data obtained in the presence of nafamostat focusing on a narrower range of y-axis values to reveal slow recovery of activity consistent with substrate-like behavior of nafamostat and reversal of inhibition. The dashed lines are projections of initial and terminal slopes to emphasize increasing rates of substrate hydrolysis as matriptase and tryptase escape effects of limiting amounts of inhibitor.

At nearly stoichiometric ratios of nafamostat to enzyme, inhibition of tryptase and β-tryptase slowly reverses, as seen in Fig 5. This behavior is consistent with nafamostat acting as a suicide inhibitor, with formation of a covalent (acyl) intermediate that is eventually broken by hydrolysis, with full release of the inhibitor and restoration of the free serine hydroxyl and enzymatic activity. The observed reversibility is not consistent with tight, non-covalent competitive inhibition, which should be stable over time, assuming that the inhibitor and protease are chemically and enzymatically stable. The rate of recovery of proteolytic activity under conditions in which the enzyme starts is in slight molar excess of nafamostat is 1–2% per hour, indicative of recovery kinetics and deacylation rates that are very slow from a pharmaceutical standpoint. Furthermore, in conditions for which there is a substantial molar excess of nafamostat, no reversibility is observed (not shown), presumably because any regenerated enzyme is promptly inactivated by formation of an acyl intermediate with a fresh molecule of nafamostat.

### Sensitive active site-titration assay of matriptase and β-tryptase using nafamostat

**Fig 6:**
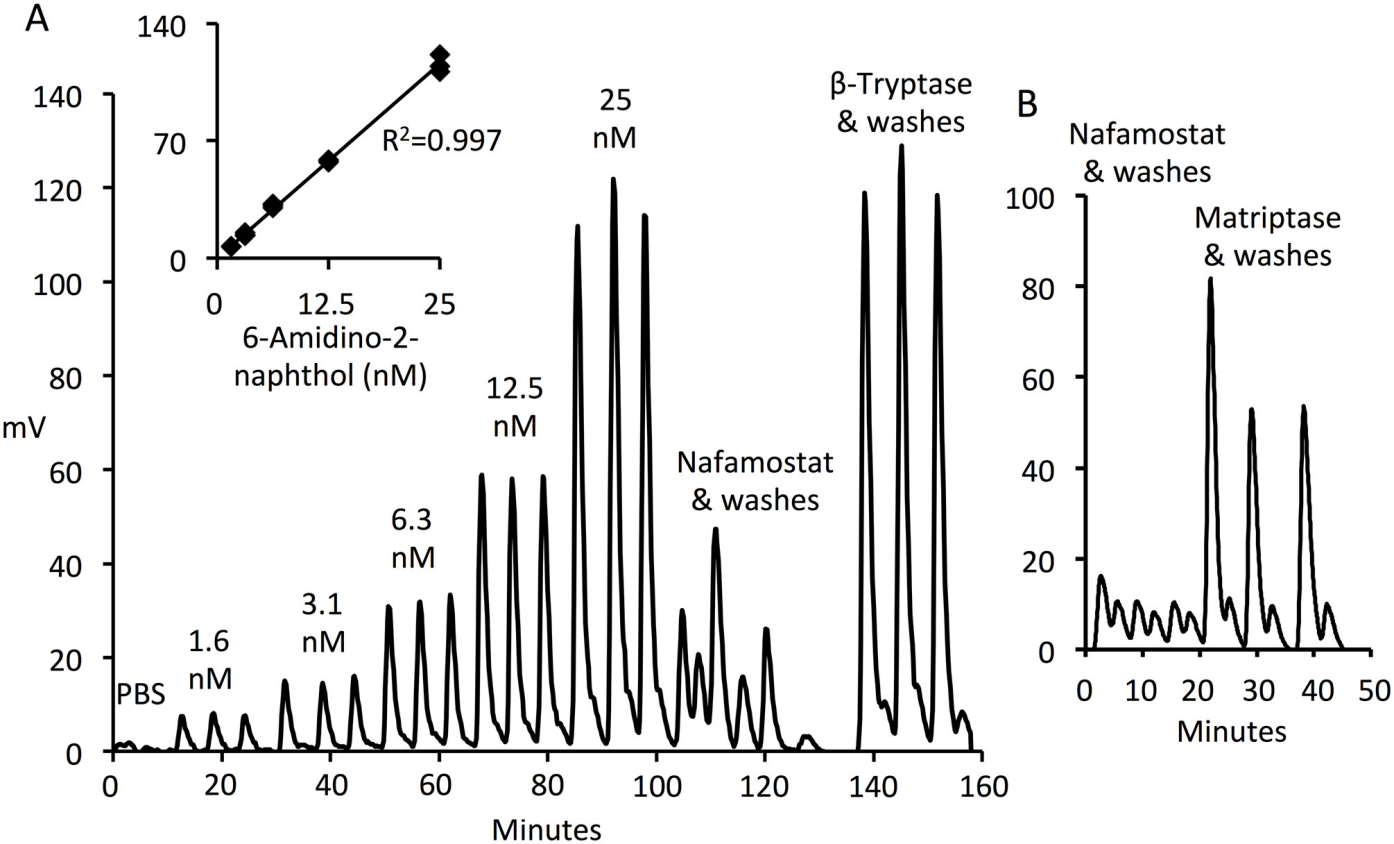
Active site titration by detecting release of 6-amidino-2-naphthol from nafamostat. Panel A shows output (in mV) from an in-line fluorescence detector generated by serial injections of 0.1-ml aliquots of a range of concentrations of 6-amidino-2-naphthol, nafamostat alone, and β-tryptase-incubated nafamostat into the flow cell of a chromatographic pump system at a flow of 0.1 ml/min of PBS to minimize bleaching. Each injection was chased by injection of an equal volume of PBS alone and each set of injections was performed in triplicate. The inset is a graph of a standard curve of fluorescence output versus concentration of 6-amidino-2-naphthol, by reference to which the active-site concentration of β-tryptase was derived. Panel B shows examples of 6-amidino-2-naphthol detected in a similar manner after incubation of nafamostat with matriptase.

Given the observed nearly 1:1 stoichiometry and slow reversal of inhibition of matriptase and β-tryptase by nafamostat, we sought to detect release of 6-amidino-2-naphthol and to explore the possibility of using nafamostat as a “burst titrant” of active sites in preparations of these enzymes. We did not assess this possibility for prostasin or HAT because inhibition for these proteases did not approach 1:1 stoichiometry. Estimates of active site concentration in our stock preparation of trypsin were similar whether obtained using the colorimetric approach with p-nitrophenyl-p’-guanidinobenzoate or the fluorometric approach with nafamostat (0.83 versus 0.62 mM, respectively). However, the nafamostat method, as implemented using the in-line flow cell of the Jasco FP-2020 Plus Fluorescence Detector, was far more sensitive, requiring dilution of trypsin stock solution ~1,000-fold. The fluorometric nafamostat method, being capable of detecting active sites in the 1-nM range (see Fig 6) using sample volumes of 0.1 ml or less, offered the prospect of minimizing consumption of enzymes that are difficult or costly to produce.

Application of the fluorometric nafamostat approach to matriptase and β-tryptase (Fig 6) suggests its utility as a sensitive method to quantify active sites. Titration of our preparation of matriptase identified an active-site concentration of 0.12 ± 0.02 μM (mean ± SD, N = 4), predicting that the preparation is 22% active using a denominator based on the manufacturer’s estimate of protein concentration. Based on the active site estimate, the specific activity of matriptase for substrate QAR using our standard assay conditions is 3 x 10^7^ Absorbance Units_410 nm_ min^-1^ M^-1^. Assay of β-tryptase active sites in similar fashion yielded an active site concentration of 2.1 ± 0.1 μM (mean ± SD, N = 3), which is 110% of the value based on the manufacturer’s estimate of protein concentration. Based on the active site estimate, the specific activity of β-tryptase for substrate GPK using our standard assay conditions is 2.5 x 10^7^ Absorbance Units_410 nm_ min^-1^ M^-1^. Nafamostat itself has minimal fluorescence at excitation and emission wavelengths optimized for 6-amidino-2-naphthol [23]. The observed low-level baseline fluorescence in stock solutions of nafamostat (as seen in Fig 6) may be due to non-enzymatic hydrolytic conversion of a small proportion of nafamostat to guanidinobenzoate and fluorogenic 6-amidino-2-naphthol.

### Inhibition of apical tryptic enzyme activity in epithelial cells cultured at an air-liquid interface

**Fig 7:**
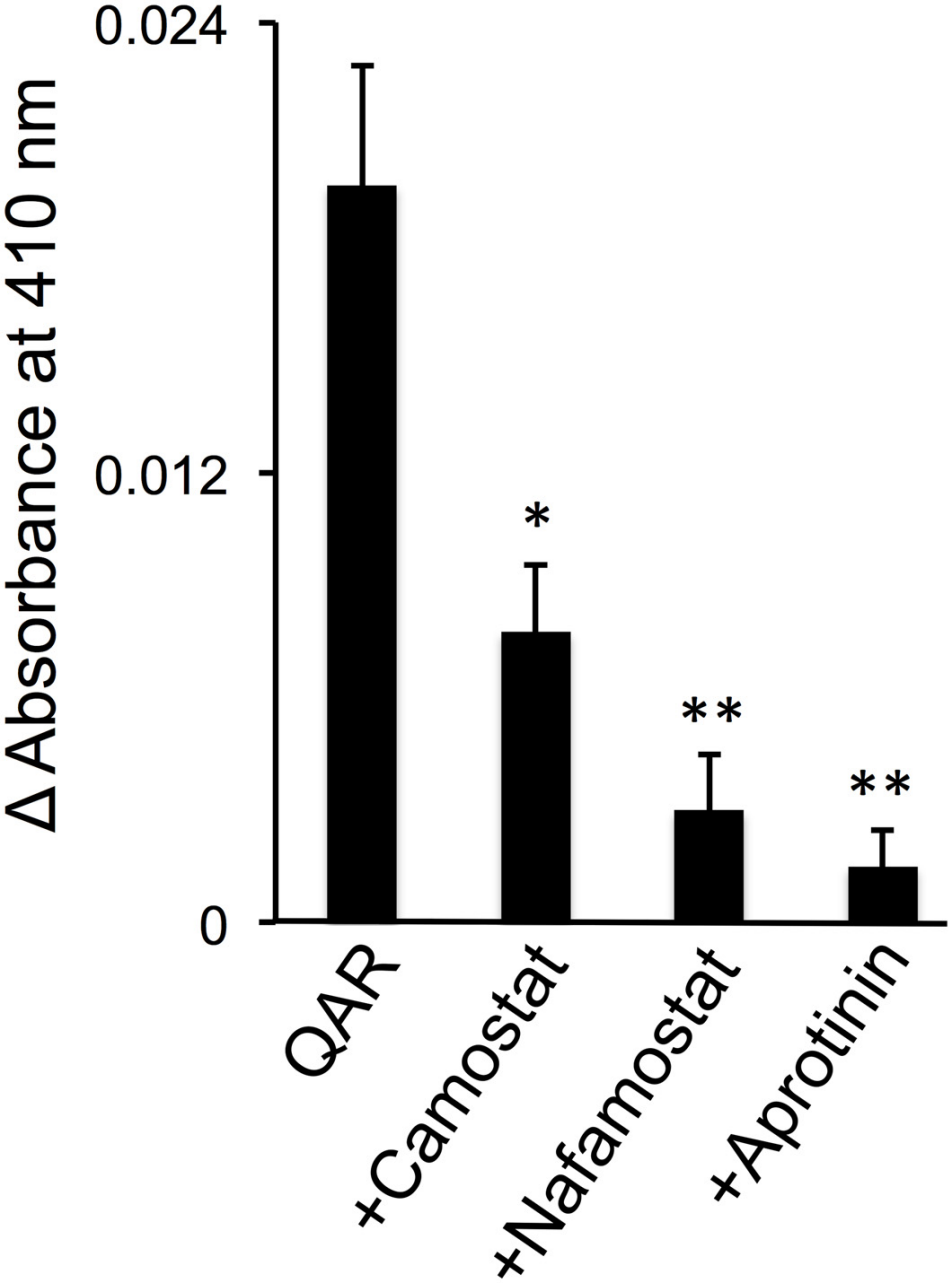
Inhibition of surface tryptic activity on bronchial epithelial cells. Monolayers of CFBE41o- cells cultured on Transwell inserts at an air-liquid interface to promote differentiation, including apical-basolateral polarization, were assayed for surface tryptic activity by adding substrate QAR to apical bathing medium, with or without addition of camostat, nafamostat or aprotinin, followed by spectrophotometric monitoring of cleaved substrate at 410 nm. *P <0.05 and **P <0.01 versus change in absorbance in QAR medium without inhibitor.

Fig 7 reveals that apical, cell surface, QAR-hydrolyzing protease activity is almost completely inhibited by aprotinin (100 μM), consistent with our prior study [3], while establishing that nafamostat and camostat (10 μM) are nearly as effective as aprotinin. In conjunction with our prior studies showing little if any active matriptase on the apical surface of CFBE41o- cells cultured in the same manner [3], the present data are consistent with prostasin (and not HAT or β-tryptase) being the principal source of apical tryptic activity.

## Discussion

This study focuses on four proteases that share three major attributes: 1) they are trypsin-like, 2) they are found in human airway epithelium and 3) they are proposed as targets for inhibition to treat allergic or infectious airway disorders associated with inflammation and hypersecretion. This first direct comparison of these proteases reveals that each has a distinct profile of susceptibility to the inhibitors shown in Fig 1, despite sharing a capacity to cleave peptides after arginine residues. Several inhibitors examined here have been used to target specific airway tryptic proteases in vivo. Although these inhibitors exhibit a broad range of potency, none is selective for any one of the proteases examined (as shown in Figs 2 and 3. Among the implications of these findings is that pathology-modifying phenotypes resulting from application of these inhibitors potentially may arise from inactivation of proteases other than those that were targeted. The findings also raise the possibility of undesired bystander effects resulting from inactivation of these and other tryptic proteases. On the other hand, some of the inhibitors, such as nafamostat for tryptase and matriptase—and aprotinin for prostasin—were exceptionally potent, raising the possibility of developing more selective inhibitors with retained potency.

In the case of β-tryptase and matriptase, the findings show that nafamostat’s high potency relates in part to actions as a suicide substrate. This results in formation of a covalently bound, inactivating intermediate that is stable for hours in aqueous solution. In this regard, nafamostat’s bifunctionality could influence potency. As shown in Fig 1, nafamostat has the potential to occupy the tryptic primary specificity pocket using either its guanidino or its amidino end, but not both simultaneously. These docking modes have different consequences. Binding via the guanidino end positions nafamostat’s carbonyl carbon to be attacked by the protease’s active site serine Oγ to yield the 4-guanidino-benzoylated “acyl” enzyme. This is a substrate-like interaction that leaves a bound fragment that cannot be competitively displaced by substrate. By contrast, docking with the amidino end in the specificity pocket is a competitive, reversible interaction that neither positions nafamostat for hydrolytic attack nor results in formation of an acyl intermediate. In the examples of matriptase and β-tryptase, the nearly 1:1 stoichiometry of inactivation by nafamostat, combined with the evidence of 6-amidino-2-naphthol release and the finding that inhibition by 6-amidino-2-naphthol itself is comparatively weak, suggest that the binding mode with the guanidino end in the primary specificity pocket is highly favored. This is less likely to be the case for HAT and prostasin, towards which nafamostat is less potent.

It can be noted from the structures in Fig 1 that camostat lacks nafamostat’s duality. Binding via its guanidino end is likely its only productive mode of action as an inhibitor, and predicts that its interactions necessarily involve formation of an acyl intermediate. However, the finding that camostat is much less potent than nafamostat as an inhibitor of matriptase and β-tryptase reveals that the mere presence of a 4-guanidino-benzoate moiety susceptible to nucleophilic attack to form a covalent intermediate does not guarantee high potency. Nonetheless, inhibition by camostat is complete at higher ratios of inhibitor to enzyme and is likely to be as durable as inhibition by nafamostat, given that the 4-guanidino benzoate moiety ends up covalently linked to prostasin to form the acyl enzyme complex captured and identified in prostasin crystallized either with camostat [25] or with nafamostat [24].

Nafamostat’s ability to form an inhibitory complex at low concentration via a cleaved intermediate is undoubtedly responsible for the report of a highly favorable (sub-nanomolar) dissociation constant (*K*_i_) when tested versus human β-tryptase [26]. However, as the present results suggest that nafamostat is a suicide substrate and burst titrant for tryptase, *K*_i_ calculations, which assume a competitive and reversible mode of inhibition, are inapplicable to nafamostat inhibition of tryptase, for which the resulting data would depend on enzyme concentration. Similar considerations apply to camostat, which yielded apparent *K*_i_ values that were >100-fold higher for prostasin than for matriptase [6], in contrast to our data in Table 2 showing that camostat is actually less potent versus matriptase than versus prostasin based on determination of concentration-normalized IC_50_, which is a more appropriate comparator for a suicide substrate. The discrepancy probably relates to the need to use concentrations of prostasin in tryptic substrate cleavage assays that are high relative to the concentrations needed of matriptase, which is an intrinsically more efficient enzyme [3]. Regardless, our data suggest that release of fluorogenic 6-amidino-4-naphthol from nafamostat by the catalytic serine of matriptase and β-tryptase is an active-site titration method that is much more sensitive than colorimetric methods. However, the findings also suggest that nafamostat will be less useful in this regard for HAT and prostasin, for which stoichiometries are less favorable.

The kinetic comparisons of prostasin, matriptase and HAT with the native, soluble β-tryptase tetramer were conducted with recombinant forms of these enzymes expressed as soluble catalytic domains. This is a matter of necessity since the assays developed to detect the activity of membrane-bound tryptic proteases are ill-suited to IC_50_ determinations because of low concentrations of surface enzyme, the need for prolonged incubations, and the presence of other peptidases in living cells. A potential caveat to the work reported here is that inhibitor susceptibility of membrane-anchored type I (prostasin) and type II proteases (matriptase and HAT) may differ from that of soluble forms of the enzymes due to active site blockade or allosteric effects. However, matriptase and prostasin can exist natively in shed, soluble forms, and HAT’s shed form may be the native enzyme’s dominant form [18]. Furthermore, the small inhibitors profiled here are unlikely to be subject to steric constraints that might limit ability of a large, proteinaceous inhibitor to inactivate a surface-bound enzyme. Indeed, even aprotinin, which is the largest inhibitor in the present study, sharply reduces apical surface tryptic activity in cultured human airway epithelial cells, which contain prostasin and matriptase in membrane-attached forms [3].

Although one of the principal findings of this study is the relative lack of inhibitor specificity towards four of the tryptic enzymes that have been considered as candidates for therapeutic inactivation in airway diseases, our data provide grounds for considering that prostasin is the principal source of apical surface tryptic activity in the human airway epithelial cell monolayers generating the data in Fig 7. The reasoning involves combining the present data generated using small inhibitors with prior studies using anti-matriptase single chain antibody [3], which is potent and selective for matriptase but inhibited only a small portion of aprotinin-sensitive tryptic activity on the apical surface of airway epithelial cells. Clearly, β-tryptases cannot be a source aprotinin-sensitive activity, because human β-tryptases completely resist aprotinin, even though aprotinin inhibits most trypsin-like serine proteases. The basis of tryptase’s unusual resistance to aprotinin is a restricted active site [36]. Compared to prostasin and matriptase, HAT is much less sensitive to aprotinin and nafamostat, but all enzymes are similarly sensitive to camostat. Thus, the data are consistent with prostasin being a major source of airway surface tryptic activity and the major source of aprotinin-sensitive activity on the apical surface of cultured airway cells.
